# Thirty years of an advanced field epidemiology training programme: a mixed-methods evaluation, Germany, 1996 to 2025

**DOI:** 10.2807/1560-7917.ES.2026.31.35.2600395

**Published:** 2026-09-03

**Authors:** Simon Brinkwirth, Lena Ella Schneider, Ida Sperle, Petra von Berenberg-Gossler, Katharina Alpers, Sofie Gillesberg Lassen

**Affiliations:** 1 Department of Infectious Disease Epidemiology, Robert Koch Institute (RKI), Berlin, Germany; * These authors contributed equally and share first authorship

**Keywords:** Program Evaluation, Epidemiology, Germany, Health Workforce, Education, Public Health Professional, Communicable Disease Control

## Abstract

**INTRODUCTION:**

Field epidemiology training programmes (FETPs) strengthen public health workforce capacity for infectious disease surveillance and outbreak response. The Postgraduate Training for Applied Epidemiology (PAE) is a 2-year FETP aligned with European and global training standards.

**AIM:**

We evaluated the contribution of PAE to a strengthened public health workforce for infection protection in Germany.

**METHODS:**

Our evaluation consisted of three components: a semi-structured desk review of programme outputs (1996–2025), an online graduate survey (cohorts 1996–2023), and an online workshop with experts from local, federal state and national health authorities. We analysed quantitative data descriptively, and qualitative data through a simplified content analysis.

**RESULTS:**

By December 2025, PAE had 126 graduates with the following outputs: 197 peer-reviewed publications, 193 accepted conference abstracts, and 205 conducted outbreak investigations. In total, 72% (91/126) graduates responded to the survey. Most respondents remained in the German public health workforce (70%; 64/91), reported high satisfaction (90%; 82/91), and would recommend the programme (96%; 87/91). Graduates reported roles in crisis and outbreak management (70%; 64/91), holding leadership roles (59%; 54/91), and engaging in knowledge dissemination (86%; 78/91). Qualitative findings highlighted shared methodological approaches and technical language, and strong networks as key facilitators of collaboration across all levels of the German public health service.

**CONCLUSION:**

We found that PAE contributes to strengthening and sustaining an infection protection workforce in Germany that regularly applies and disseminates learnt skills. Most graduates have held or currently hold leadership roles and frequently contribute to outbreak and public health crisis management.

**Key public health message**

**What did you want to address in this study and why?**

Evidence on the long-term impact of Field Epidemiology Training Programmes (FETP), particularly on workforce retention, remains limited and is needed to better highlight their importance for resilient public health systems. We evaluated the German FETP 30 years after its establishment to assess its contribution to infection protection capacity and the public health workforce.

**What have we learnt from this study?**

The programme builds and retains a skilled public health workforce, with over 70% of graduates working in public health services in Germany. Graduates are frequently involved in outbreak response and surveillance, also taking on leadership roles, while sharing expertise across the system. The findings confirm the value of the programme in developing core competencies and strong professional networks.

**What are the implications of your findings for public health?**

Field epidemiology training programmes are essential for strengthening public health systems. They enable collaboration across institutions through shared methodological approaches and professional networks. Expanding their reach across all levels of the public health system, particularly at regional and local levels, could further strengthen capacity.

## Introduction

Field epidemiology training programmes (FETPs) are a key component to strengthen public health workforce for infectious disease surveillance as well as outbreak investigation and management [1]. The need for trained infectious disease epidemiologists has become increasingly evident through major public health challenges such as the COVID-19 pandemic and rising antimicrobial resistance [2,3]. The first 2-year FETP, the Epidemic Intelligence Service, was established by the United States Centers for Disease Control and Prevention (US CDC) in 1951. Since then, FETPs have been established worldwide and expanded with a possible three-tiered FETP model: basic, intermediate and advanced [1,4,5].

The 2-year German advanced FETP named Postgraduate Training for Applied Epidemiology (PAE) was established in 1996 at the Robert Koch Institute (RKI) with initial support from the US CDC [6]. Its overall aim is to build and retain a skilled public health workforce through a high-quality FETP to strengthen infectious disease prevention and control across public health services in Germany. The PAE has a learning-by-service approach with only 10–15% of time spent in in-person or online teaching modules with minimum travel of 8 weeks over 2 years [7,8]. Initially, all PAE participants, commonly referred to as fellows, were hosted and funded by the Department of Infectious Disease Epidemiology at the RKI. In 2006, PAE expanded its training sites to include federal state health offices and, from 2021, larger local health authorities [8]. Since 2021, PAE fellows have been funded by their training site. While a fixed number of fellowship positions are advertised by the RKI each year, PAE is also used as a capacity and qualification mechanism for staff holding positions, particularly at local and federal state level. Selection criteria include a health-related postgraduate degree and relevant work experience. From 2009, PAE fellows could simultaneously be enrolled and obtain a Master of Science in Applied Epidemiology degree at the Charité – Universitätsmedizin Berlin.

The PAE collaborates globally through the Training Programs in Epidemiology and Public Health Interventions Network (TEPHINET). The PAE is one of the founding members of TEPHINET and received its TEPHINET accreditation in 2019 [5]. Concurrently with PAE and in collaboration with the RKI, the European Programme for Intervention Epidemiology Training (EPIET) was established in 1995 [9]. The European Centre for Disease Prevention and Control (ECDC) has been hosting EPIET for the European Union (EU) countries since 2007 [10,11]. The PAE is associated with the ECDC Fellowship Programme’s EPIET path, which entails that PAE and EPIET fellows follow the same curriculum in English, including formal teaching modules, field assignments (outbreak management, surveillance, public health research, teaching and communication), progress monitoring tools and graduation criteria, which are all described in the EPIET manual [12]. In addition, the PAE fellows attend a laboratory module at the RKI, follow weekly scientific seminars and gain experience at local health authorities through a 1-week internship. While most training modules are organised by the ECDC, PAE contributes with their organisation, and provides expert time towards teaching, facilitation, teaching methods and individual fellow supervision. Both PAE and EPIET are historically linked with the ECDC European Scientific Conference on Applied Infectious Disease Epidemiology (ESCAIDE) [13]. Although submitted abstracts are not accepted by default, it is required that PAE fellows submit at least two abstracts to and attend the ESCAIDE conference during their fellowship [12]. Within the EU, PAE also collaborates with the Greek-FETP and the UK-FETP to enhance the network between FETPs and cover competencies not included in the EPIET curriculum.

Although some quality assurance of PAE is given through its TEPHINET accreditation, accreditation is limited to assessing current fellows and graduates from the last 2 years. At 30 years of existence, PAE outputs have not yet been described in full nor has PAE been evaluated in terms of whether it achieves its aim of strengthening infectious disease prevention and control across all levels of the German federal public health services, namely local, federal state and national level. We aimed to conduct a mixed-methods evaluation of the PAE 30 years after its initiation to address these gaps and inform future programme developments.

## Methods

We applied a mixed-methods design comprising three complementary components: a semi-structured desk review, an online survey among PAE graduates and an online workshop with PAE alumni and supervisors from national, federal state and local health authorities. The evaluation was structured around three programme objectives: developing and retaining a skilled public health workforce, providing a high-quality applied FETP, and strengthening infectious disease prevention and control through the application and transfer of epidemiological expertise. These objectives were assessed across six evaluation attributes (Table 1). Curriculum content and organisational aspects were beyond the scope of this evaluation.

**Table 1:** Mapping of the Postgraduate Training for Applied Epidemiology programme objectives, evaluation attributes and data sources, Germany, November 2025–March 2026

Programme objective	Evaluation attribute	What was assessed?	Data source
Develop and retain a skilled public health workforce for infectious disease prevention and control in Germany	Programme outputs	Number of fellows and graduates; distribution across training sites; scientific outputs; supported outbreak investigations	Desk review
	Recruitment profile and retention	Educational background; retention in the German public health service; current employment; leadership roles	Graduate survey; expert workshop
Provide a high-quality applied field epidemiology training programme	Satisfaction and perceived programme value	Overall satisfaction; willingness to recommend the programme; perceived value for professional development; satisfaction with programme components	Graduate survey; expert workshop
	Competencies	Competencies developed through the programme; additional competencies gained; perceived competency gaps	Graduate survey; expert workshop
Strengthen infectious disease prevention and control through the application and transfer of epidemiological expertise	Knowledge application, dissemination and transfer	Application of acquired competencies; frequency and forms of knowledge and skills sharing	Graduate survey; expert workshop
	Outbreak and crisis management	Involvement in outbreak and crisis management after graduation; roles and responsibilities during responses; perceived preparedness for future crises	Graduate survey; expert workshop

### Desk review

For the semi-structured desk review, we collated information from 1996 to 2025 on (i) the total number of PAE fellows who have started the fellowship since its beginning by training site, (ii) the number of outbreaks with PAE fellows as lead investigator going through all steps of an outbreak investigation [12], (iii) the number of peer-reviewed publications with PAE fellows as co-author or first author based on work conducted during their fellowship, and (iv) the number of ESCAIDE presentations by PAE fellows or graduates based on fellowship projects.

To retrieve the number of peer-reviewed publications, we conducted a search in Scopus on 3 February 2026 with 'Postgraduate Training for Applied Epidemiology' or 'Field Epidemiology Training Programme, Germany' as affiliation, and screened titles and authors to ensure eligibility for inclusion. Additional name-based searches were done for fellows with fellowship starting between 1996 and 2006 during which time RKI was used as affiliation in publications rather than PAE. We identified the number of presentations at ESCAIDE conferences from abstract books available on the ESCAIDE website (2009–2025).

### Survey among graduates of the Postgraduate Training for Applied Epidemiology programme

The survey targeted all PAE graduates from the cohorts 1996 to 2023, referring to the starting year of each 2-year programme. The survey was administered in German using an online survey tool (Voxco) and remained open from 25 November 2025 to 31 January 2026.

Recruitment was conducted via the RKI all-employees mailing list, a mailing list of the federal state training sites, the EPIET Alumni Network (EAN) newsletter and the EAN booth at ESCAIDE 2025, and through snowball sampling via informal communication channels including social media and emails directed to graduates before 2008.

The survey was developed by the PAE coordination team, drawing on the thematic areas and conceptual framework of the ECDC Fellowship Programme Manual and ECDC post-graduation survey [12]. The survey was piloted with four graduates and comprised 22 questions. The full graduate survey can be found in the Supplement.

Only complete surveys were analysed and described by calculating absolute and relative frequencies. Unless otherwise stated, proportions were calculated using all survey respondents, or the relevant subgroup for conditional items, as the denominator. Missing responses including 'no answer' selections were retained in the denominator. Data analysis and figures were conducted using R (version 4.5.1).

### Expert workshop

We conducted a 2.5 h online workshop on 24 March 2026 with purposively selected PAE alumni and supervisors to contextualise preliminary findings from the desk review and graduate survey. Participants were selected to achieve balanced representation across geographical location and site level (national, federal state or local). Invitations were sent via email, with a target sample size of 10–15 participants. Participants discussed preliminary survey findings in plenary and small groups using a predefined topic guide. A workshop format was chosen to allow for exchange between different perspectives and because the topic was not considered personal or sensitive. Participants were informed at the beginning of the workshop about the objectives, their role as programme experts, voluntary participation and anonymised reporting.

A notetaker summarised plenary and group discussions on a shared Miro board allowing for real-time feedback. Discussions were not audio-recorded. The notes were analysed in MAXQDA using a simplified framework-based content analysis, with one person coding using a predefined list of codes. Additional codes were added during the analysis as new themes emerged from the data. Afterwards, the coded text was organised into a matrix for each attribute to compare and contextualise corresponding survey findings. The agenda, full list of codes and the discussion guide are provided in the Supplement. 

## Results

Of 126 PAE graduates, 91 completed the survey, resulting in a response rate of 72%. We received responses from graduates of all cohorts between 1996 and 2023, with response proportions varying by cohort and training site type. A more detailed description of respondents is included in Supplementary Table S3. We invited 20 experts to participate in the workshop, and 14 agreed: four working at the RKI, six at federal state training sites, and four at local health authorities.

### Programme outputs

By 31 December 2025, 154 individuals in 30 yearly cohorts had started the PAE fellowship and 126 of them had graduated. Of the remaining 28 fellows, 14 are fellows of current cohorts 2024 and 2025, seven are from previous cohorts with delays in finalising the fellowship, and seven ended the fellowship without graduating. The few fellowship discontinuations were primarily attributed to personal reasons, including competing job opportunities. Excluding current and delayed fellows, 95% (126/133) of PAE fellows graduated. Among all fellows and graduates, the RKI has been the training site for 71% (109/154) of fellows. Since 2006, fellows have been hosted at training sites in 12 of the 16 German federal states. The cumulative number of fellows hosted at federal state or local level training sites ranges from one to nine, with most graduates at the federal state level in Baden-Württemberg and Bavaria (Table 2).

**Table 2:** Postgraduate Training for Applied Epidemiology programme outputs: fellows per federal state, 1996–2025, Germany (n = 154)

Training site	n
National level (RKI)	109
Federal state level (total)	45
Brandenburg	1
Berlin	5
Baden-Württemberg	9
Bavaria	9
Hamburg	1
Hesse ^a^	4
Lower Saxony	5
North Rhine-Westphalia ^a^	2
Rhineland-Palatinate	6
Saarland	1
Saxony-Anhalt	1
Thuringia	1

RKI: Robert Koch Institute.

^a^ Training sites at federal state level and at local health authority.

For scientific output, we identified 197 peer-reviewed publications (published between 1 January 1997 and 3 February 2026), and 193 presentations (oral and poster) at ESCAIDE conferences between 2009 and 2025 with an average of 12 accepted abstracts per year. A total of 205 outbreak investigations including 39 COVID-19 outbreaks were supported by fellows (data available between 1 January 2009 and 31 December 2025).

### Recruitment profile and retention

Based on the graduate survey, the most common educational backgrounds before the PAE were medicine (47%; 43/91), public health (42%; 38/91), natural sciences (21%; 19/91), epidemiology (13%; 12/91) and veterinary medicine (5.5%; 5/91) (multiple answers possible). Notably, 35% (32/91) of graduates held postgraduate qualifications in more than one discipline before entering PAE. The most common combination was medicine and public health (14/32). Some workshop participants noted that the added value of PAE was higher for individuals without a second master’s degree in public health or epidemiology. At federal state and local level, workshop participants noted that identifying fellowship candidates may be challenged by PAE requirements including field assignments, English proficiency, willingness to travel, and partly perceived limited applicability of competencies acquired through the PAE to everyday work after graduation.

Most graduates (70%; 64/91) currently work in the German public health services. Employment was reported at the national (48%; 44/91), federal state (19%; 17/91) and local (4%; 4/91) level, with one graduate reporting employment at two levels (Table 3). Since graduation, 59% (54/91) of graduates have had at least one leadership role. Among 54 graduates with leadership experience, 44 reported leading a team, 33 managing projects or studies, and 17 leading a department or institution (multiple responses possible).

**Table 3:** Previous educational background and current employment of Postgraduate Training for Applied Epidemiology programme, 1996–2025, Germany (n = 91)

	n	%
Previous educational background		
Medicine	43	47
Public health	38	42
Natural sciences	19	21
Epidemiology	12	13
Veterinary medicine	5	5
Other	7	8
Response missing	4	4
Current employment		
German public health sector – national level	44	48
German public health sector – state level	17	19
German public health sector – local level	4	4
Other German institute or ministry	4	4
University or research institute	7	8
International public health organisation	3	3
Other sectors	15	16

Multiple responses were possible for previous educational background and current employment; percentages therefore do not add up to 100%.

### Satisfaction and perceived programme value

The graduate survey showed an overall high satisfaction with PAE, with 90% (82/91) reporting being very or rather satisfied and 96% (87/91) reporting they would definitely or probably recommend PAE to colleagues. The PAE was considered beneficial for the professional development with 90% (82/91) rating it as very or rather helpful. Workshop participants further detailed how PAE had been instrumental for their professional development.

**Figure 1:**
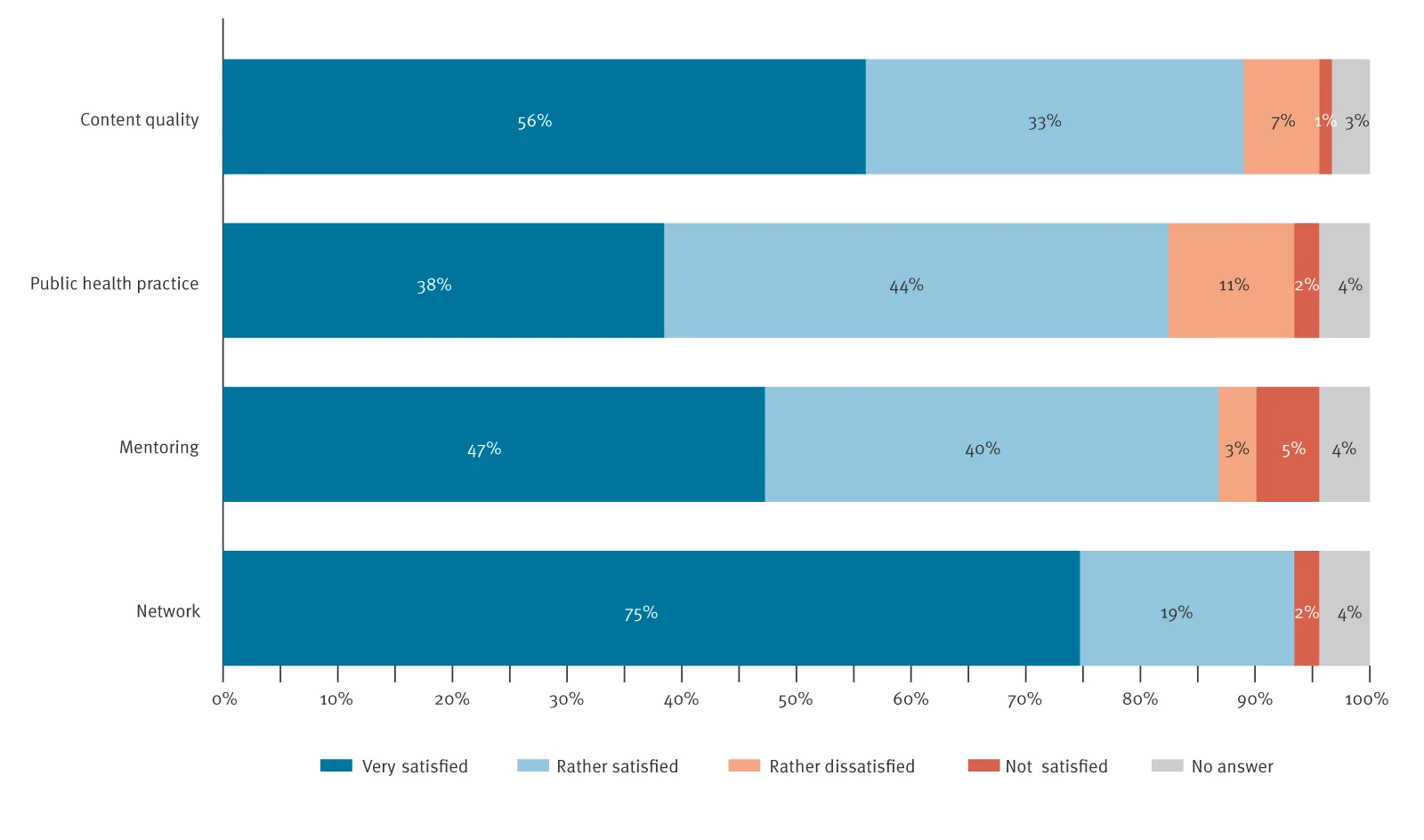
Programme satisfaction of graduates, Postgraduate Training for Applied Epidemiology evaluation survey, November 2025–January 2026, Germany (n = 91)

Looking at satisfaction across PAE components (content, public health practice, mentoring and supervision, and networking), overall satisfaction was high, with between 82% (75/91) and 98% (89/91) of respondents reporting being very or rather satisfied. The highest satisfaction was observed for international networking opportunities, with 75% (68/91) very satisfied (Figure 1). Networking also emerged as a central theme during the workshop. Workshop participants explained that interpersonal relationships facilitated collaboration both internationally and between all three levels of the public health service in Germany. Further, workshop participants mentioned that topics such as outbreak investigations and surveillance are not or only peripherally covered in academic programmes, often missing an applied perspective. The PAE was also perceived as enabling those with previously more specialised profiles to broaden their expertise.

### Competencies

**Figure 2:**
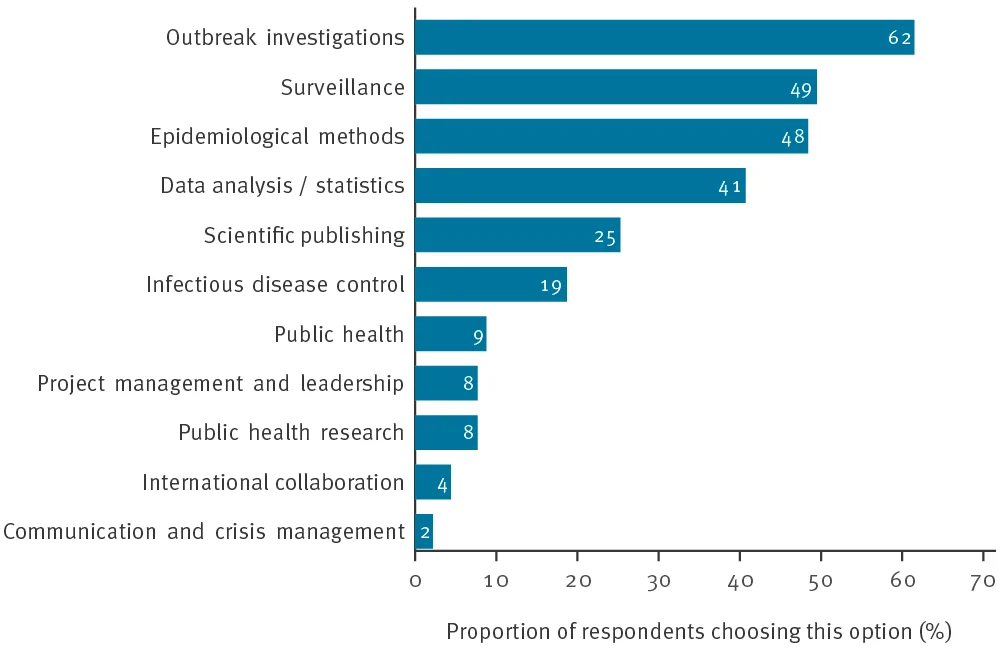
Most frequently reported acquired competencies (top three), Postgraduate Training for Applied Epidemiology evaluation survey, November 2025-January 2026, Germany (n = 91)

In the graduate survey, the three competencies most frequently reported as developed through the PAE were outbreak investigations with 62% (56/91), surveillance with 49% (45/91) and epidemiological methods with 48% (44/91). Competencies related to project management and leadership (8%; 7/91), public health research (8%; 7/91), international collaboration (4%; 4/91) and communication and crisis management (2%; 2/91) were reported less frequently (Figure 2). Workshop participants explained the benefit of the developed competencies in outbreak investigations, surveillance and epidemiological methods with the development of a common language and methodological understanding facilitating collaboration in Germany and internationally with European colleagues. Other gained competencies mentioned by workshop participants included structured work under pressure, scientific writing and communication, inter- and transdisciplinary work, and statistical programming. Competencies that workshop participants identified as not sufficiently acquired through the PAE included decision-making, accountability and sound understanding of the legal basis for infection protection in Germany. Moreover, fellows with a medical background reported loss of clinical competencies.

Participants were asked to name the three competencies they most strongly developed during the fellowship. Percentages are based on the total number of respondents.

### Knowledge application, dissemination and transfer

In the graduate survey, 37% (34/91) reported fully applying competencies acquired during the PAE in their current role, 54% (49/91) partially, and only 4.4% (4/91) hardly or not at all. In total, 45% (41/91) reported regularly sharing programme-related knowledge or skills. Among graduates who reported any knowledge sharing, the most reported forms of dissemination were exchange and discussions within the workplace (92%; 72/78), mentoring or supervising colleagues (73%; 57/78), and delivering trainings or workshops, or contributing to teaching activities (72%; 56/78). Scientific publications were mentioned by 49% (38/78) as a dissemination pathway (multiple responses possible). Workshop participants described dissemination of learnt competencies as widespread through teaching, workshops or training offers such as the Epikurs@RKI, a short-format education in key epidemiological competencies for local health authorities [14].

### Outbreak and crisis management

In total, 70% (64/91) of graduates reported having had a role in crisis or outbreak management since completing PAE; 62% (56/91) had done so multiple times, while 25% (23/91) had not taken on such a role. The three most frequently reported responsibilities in outbreak management after graduation were epidemiological analysis (75%; 48/64), leadership or coordination tasks (69%; 44/64), and field work or operational response activities (63%; 40/64). Communication with the public or media was reported by 41% (26/64) (multiple responses possible). Feeling well or very well prepared for future crisis situations because of PAE was reported by 75% (68/91), while 21% (19/91) felt hardly or not prepared. Workshop participants provided contextual insights by mentioning that graduates often function as multipliers who can build bridges through collaboration with, for example, microbiological and clinical colleagues. Workshop participants also highlighted that graduates and fellows strengthened the application of analytical epidemiological methods in outbreak investigations, contributing to a shift from primarily laboratory-based outbreak investigations towards greater use of epidemiological methods. However, workshop participants noted barriers such as concerns about operational demands and supervision which at times limited meaningful integration of fellows into larger outbreak investigations and other public health crises.

## Discussion 

This mixed-methods evaluation provides quantitative and qualitative insight into the contribution of the German FETP, the PAE, to building and retaining a skilled field epidemiology workforce supporting infectious disease surveillance, outbreak response and public health practice in Germany. This evaluation goes further than accreditations and other FETP evaluations, which have primarily focused on outputs, while evidence on factors such as retention of FETP graduates in public health services remained limited [9,15,16].

Productivity of fellows is reflected in numerous publications, ESCAIDE conference contributions and participation in outbreak investigations. While PAE graduation requires submission of at least one scientific article and presentation of one abstract at an international scientific conference, preferably ESCAIDE [12], the fellows' involvement in public health actions is documented in outbreak reports. Examples illustrate fellows in lead roles in outbreak investigations with public health impact, where they contributed to the identification of food-borne outbreak vehicles [17,18] and public health measures such as ward closures [19].

Retention of graduates within the German public health workforce was high, particularly at national and federal state level, and graduates reported taking on leadership roles. Similar observations have been reported from other advanced FETPs [1,20]. These findings should not be interpreted as evidence that the PAE itself caused workforce retention and leadership roles. Candidates are likely to have entered PAE with a strong commitment to public health, and graduates who remained professionally engaged may have been more likely to participate in the survey. Similarly, the high level of qualifications among PAE fellows probably contributed to them working in leadership positions.

In line with EPIET competencies, the main competencies developed in the PAE were: outbreak investigation, surveillance, and epidemiological methods, a strength of the learning-by-service model in developing applied epidemiological skills [21]. The practice-oriented nature and the opportunity for hands-on application of epidemiological methods distinguish FETP training from academic epidemiology programmes [22]. Some graduates nevertheless reported that they felt only partly prepared for future public health emergencies. Although the evaluation was not designed to assess individual curriculum components, this indicates that advanced FETPs need to continuously adapt to increasingly complex emergency responses and evolving public health challenges. The workshop identified gaps in accountable decision-making, which may be less readily developed in a closely supervised programme such as the PAE.

The PAE’s contribution extends beyond developing individual competencies in that it strengthens professional networks and facilitates the dissemination of epidemiological expertise. The number of fellows hosted by training sites in the federal states has been rising continuously since its introduction in 2006, with 12 of 16 German federal states having or having had an active PAE training site. Networking and a common technical language contribute to strengthening collaboration between public health service levels and across disciplines. Competencies acquired during the fellowship are amplified as graduates share knowledge within their organisations through mentoring colleagues, supervising trainees and teaching. Similar effects have been described in international evaluations of FETPs [22,23].

Only four graduates reported working at the local level. This finding is relevant for future workforce development and may reflect both differences in workforce needs and graduates' preference for specialised epidemiological roles. Advanced FETP graduates are more likely to pursue positions at the national or federal state level, where the competencies acquired during the fellowship may more closely align with specialist functions. Tiered training approaches that combine advanced programmes with more accessible basic and intermediate FETPs may more adequately address workforce needs across all levels [4,20]. Such an approach is currently under development in Germany [24]. Funding costs and programme requirements such as English proficiency, time away from home due to travel and formal training may also be barriers to identifying motivated suitable candidates among staff at federal state and local level.

The high survey response rate and representation of graduates from almost all PAE cohorts support the robustness of our findings. Nevertheless, several limitations should be considered. The evaluation was conducted in close collaboration with the PAE leadership and team. While the PAE leadership did not participate in the expert workshop, the evaluation was not an independent external assessment. We may have underestimated some programme outputs: firstly, historical outbreak records were incomplete. Secondly, the literature search relied on programme affiliation, and some journals allow only a single institutional affiliation per author. Moreover, fellows not consistently using PAE as an affiliation, especially before 2006, prevented a standardised search. Thirdly, we only considered conference contributions to ESCAIDE, as an important conference for PAE. Fourthly, our survey recruitment may have overestimated workforce retention, programme satisfaction and perceived programme value. Fifthly, workshop participants were selected for their experience with PAE either as alumni or supervisors. While close knowledge of the programme was necessary to provide insight, this is likely to have resulted in positive bias. Finally, a simplified framework-based analysis conducted by a single coder may have increased researcher subjectivity and reduced reliability.

## Conclusion

Over the past three decades, the German advanced FETP, PAE, has contributed to the development of a skilled workforce for infectious disease prevention and control, with a high proportion of graduates continuing to work within the public health system at national and federal state level. The PAE graduates report continued application and transfer of epidemiological expertise including in leadership positions. The programme's contribution appears to extend beyond individual graduates as training site and graduate networks facilitate collaboration through a common technical language. Complementary basic and intermediate training pathways may help strengthen epidemiological capacity at local level in Germany's public health system.

## Data Availability

Data are available upon request from corresponding author.

## Supplementary Data

268000 Supplement
