# Supplementary material for: Thirty years of an advanced field epidemiology training programme: a mixed-methods evaluation, Germany, 1996 to 2025
Source: Euro Surveill. 2026 Sep 3;31(35):2600395. doi: 10.2807/1560-7917.ES.2026.31.35.2600395 (PMC13545520; doi:10.2807/1560-7917.ES.2026.31.35.2600395)
Supplement: Supplementary file 1 [file 26-00395_GILLESBERG_Supplement.pdf]

Supplementary Material

Title: Thirty Years of an Advanced Field Epidemiology Training Programme in Germany: A Mixed-Methods Evaluation

Authors: Simon Brinkwirth, Lena Ella Schneider, Ida Sperle, Petra von Berenberg-Gossler, Katharina Alpers, Sofie Gillesberg Lassen

This supplementary material is hosted by Eurosurveillance as supporting information alongside the article „Thirty Years of an Advanced Field Epidemiology Training Programme in Germany: A Mixed-Methods Evaluation ", on behalf of the authors, who remain responsible for the accuracy and appropriateness of the content. The same standards for ethics, copyright, attributions and permissions as for the article apply. Supplements are not edited by Eurosurveillance and the journal is not responsible for the maintenance of any links or email addresses provided therein.

Content

Supplementary Table S1: Agenda of PAE Evaluation expert workshop, April 2026, Germany 2

Supplementary Table S2: Codes for simplified framework-based content analysis, PAE Evaluation expert workshop, April 2026, Germany 3

PAE Evaluation expert workshop discussion guide: Breakout group and plenary discussion topics 4

Supplementary Table S3: Response proportion by training site and cohort, Postgraduate Training for Applied Epidemiology evaluation survey, November 2025-January 2026, Germany (n = 91) 5

Supplementary Table S1: Agenda of PAE Evaluation expert workshop, April 2026, Germany

Time	Format	Topic	Description
2:20-2:30	Plenary	Arrival	Welcome, technical check, and introductory chat activity
2:30-2:35	Plenary	Welcome	Introduction of the evaluation team and workshop objectives
2:35-2:40	Plenary	Objectives	Presentation of the workshop aims, framing, and intended use of the evaluation findings
2:40-2:45	Plenary	Agenda	Overview of the workshop agenda and discussion guidelines
2:45-2:55	Plenary	Survey results	Presentation of the first results of the PAE evaluation
2:55-3:00	Plenary	Questions	Clarification questions regarding the presented results
3:00-3:50	Breakout groups	Group discussion	Structured discussions guided by a moderator and documented on a Miro board
3:50-4:05	Plenary	Break	Short break
4:05-4:20	Plenary	Feedback group discussions	Presentation key findings and discussion points from groups
4:20-4:25	Plenary	Questions	Clarification and discussion
4:25-4:35	Plenary	Interactive poll	Slido poll on the future development of the PAE programme
4:35-4:55	Plenary	Open plenary discussion	Discussion of future adaptations to strengthen the programme
4:55-5:00	Plenary	Closing	Summary, next steps, and acknowledgements

Supplementary Table S2: Codes for simplified framework-based content analysis, PAE Evaluation expert workshop, April 2026, Germany

**List of codes**	**Category**
Perceived programme value	predefined
Networking	predefined
Common language	added during analysis
Satisfaction	predefined
Career relevance and advancement	predefined
Practical applicability	added during analysis
Varying added value by system level	predefined
Post-programme workforce contribution	predefined
Leadership positions	predefined
Knowledge dissemination and multiplier effects	predefined
Application in outbreak/crisis roles	predefined
Competency development	predefined
Competency gaps	predefined
Core competencies	predefined
Participant profile and retention	predefined
Highly qualified entry profile	predefined
Retention in public health service	predefined
Concentration at national/regional level	added during analysis

PAE Evaluation expert workshop discussion guide: Breakout group and plenary discussion topics

Topic 1: Contribution of the PAE programme to strengthening infectious disease control (all groups)

Participants discussed the impact of the programme on professional expertise at:

- national level,
- federal state level, and
- local public health authority level.

Follow-up questions explored where the programme's added value was perceived to be greatest and where potential gaps remained.

Topic 2: Contribution to professional development (Group A)

Participants discussed:

- the role of the programme in their own professional development or that of fellows they had supervised; and
- whether certain professional profiles benefited particularly from the programme and which aspects should be considered during fellow selection.

Topic 3: Competency development and application (Group B & C)

Group B – Competency development

- Which competencies are most strongly developed through the fellowship?
- Which competencies receive comparatively less attention?

Group C – Knowledge application and transfer

- To what extent are competencies acquired during the fellowship applied and disseminated in everyday professional practice?

Plenary discussion on the future of the PAE programme

- What adaptations are needed to increase the number of PAE graduates working across different levels of the German public health service and thereby strengthen infectious disease control?
- How can competencies acquired during the PAE programme be disseminated more broadly throughout the public health service?
- Which mechanisms could facilitate knowledge transfer and capacity building?

Supplementary Table S3: Response proportion by training site and cohort, Postgraduate Training for Applied Epidemiology evaluation survey, November 2025-January 2026, Germany (n = 91)

	Total (N)	Respondents (n)	Response proportion (in %)
Training site			
National levels graduates	90	59	66
State and local level graduates	36	29	81
NA		3	-
Cohorts			
1996–1999	7	2	29
2000–2003	9	3	33
2004–2007	19	10	53
2008–2011	21	15	71
2012–2015	19	16	84
2016–2019	20	16	80
2020–2023	31	23	74
NA		6	-

Postgraduate Training for Applied Epidemiology evaluation survey, 2025, Germany

Background and career paths

1. In which period did you start the programme?

1996–1999; 2000–2003; 2004–2007; 2008–2011; 2012–2015; 2016–2019; 2020–2023; no response

1b. In which institution were you based during your fellowship?

Robert Koch Institute (RKI); Federal state authority; Local public health authority; no response

2. In which field did you hold a degree prior to starting the programme?

(multiple responses possible)

Human medicine; Epidemiology; Public health; Natural sciences (e.g. biology, microbiology); Veterinary medicine; Other (please specify); no response

3. What was your highest academic qualification prior to starting the programme?

Bachelor's degree; Master's degree / Diploma; State examination; Doctorate (PhD / Dr rer nat / Dr med); Other (please specify); no response

4. What was your first position after completing the programme?

(multiple responses possible)

Local public health authority (Germany); State health authority or state institute of public health (Germany); Robert Koch Institute; Other national institute or federal ministry (Germany); National public health institute abroad; International public health organisation (e.g. European Centre for Disease Prevention and Control, World Health Organization, European Food Safety Authority, Food and Agriculture Organization); University or research institution (Germany); University or research institution (abroad); Non-profit organisation / non-governmental organisation (NGO, foundation); Pharmaceutical company; Private sector company outside the pharmaceutical industry (e.g. consultancy or health services company); Hospital or clinical setting; Laboratory; Self-employed / freelance; Enrolled as a doctoral student (PhD); Other (please specify); no response

5. Where are you currently employed?

(multiple responses possible)

Local public health authority (Germany); State health authority or state institute of public health (Germany); Robert Koch Institute; Other national institute or federal ministry (Germany); National public health institute abroad; International public health organisation (e.g. European Centre for Disease Prevention and Control, World Health Organization, European Food Safety Authority, Food and Agriculture Organization); University or research institution (Germany); University or research institution (abroad); Non-profit organisation / non-governmental organisation (NGO, foundation); Pharmaceutical company; Private sector company outside the pharmaceutical industry (e.g. consultancy or health services company); Hospital or clinical setting; Laboratory; Self-employed / freelance; Enrolled as a doctoral student (PhD); Other (please specify); no response

6. What is your current role?

(multiple responses possible)

Epidemiologist; Public health specialist; Microbiologist / laboratory professional; Medical doctor / clinical role; Leadership / management role; Other (please specify); no response

Programme experiences

7. Please rate the following aspects of the programme:

	Not satisfied	Slightly satisfied	Moderately satisfied	Very satisfied
Quality of technical training	☐	☐	☐	☐
Practical relevance to public health services	☐	☐	☐	☐
Supervision / mentoring	☐	☐	☐	☐
International networking (e.g. European Programme for Intervention Epidemiology Training, Training Programs in Epidemiology and Public Health Interventions Network)	☐	☐	☐	☐
Opportunity to obtain a Master’s degree	☐	☐	☐	☐

8. How helpful was the programme for your professional development?

	Not helpful	Slightly helpful	Moderately helpful	Very helpful
Programme contribution to professional development	☐	☐	☐	☐
MSAE (Master’s degree) contribution to professional development	☐	☐	☐	☐

9. Which competencies did you most strongly develop through the programme?

(up to three responses)

Epidemiological methods; Data analysis / statistics; Public health; Infectious disease control; Outbreak investigations; Public health research; Surveillance; Project management and leadership; Scientific publishing; Communication and crisis management; International collaboration; Other (please specify); no response

10. Are you able to apply the competencies acquired during the programme in your current role?

Yes, fully; Yes, partially; No, hardly; No, not at all; no response

11. What role does the EAN alumni network currently play for you?

Very important; Moderately important; Less important; Not important; no response

Knowledge exchange

12. Have you shared knowledge or skills acquired through the programme with colleagues after your graduation?

Yes, regularly; Yes, occasionally; No, hardly; No, not at all; no response

13. In which ways have you shared knowledge acquired through the programme?

(multiple responses possible)

Trainings / workshops / module facilitation; Mentoring / supervision; Scientific publications; Internal exchange / discussions within the workplace; Other (please specify); no response

Crisis and outbreak management

14. Since completing the programme, have you taken on a role in crisis or outbreak management?

Yes, multiple times; Yes, once; No; no response

15. Which role(s) have you taken on in crisis or outbreak management?

(multiple responses possible)

Epidemiological analysis; Leadership / coordination; Communication with the public / media; Fieldwork / operational response; Other (please specify); no response

16. Do you feel that the programme has prepared you for future crisis situations?

	Not at all prepared	Slightly prepared	Well prepared	Very well prepared
Preparedness for crisis situations	☐	☐	☐	☐

Leadership and responsibilities

17. Since completing the programme, have you taken on leadership responsibilities?

Yes; No; no response

18. If yes: What type of leadership responsibilities have you taken on?

(multiple responses possible)

Project / study leadership; Team leadership; Leadership of a department or institution; Other (please specify); no response

Overall satisfaction

19. Have you already recommended, or would you recommend, the programme to colleagues?

Yes, definitely; Yes, probably; Probably not; No; no response

20. Overall, how satisfied are you with the programme?

	Not satisfied	Slightly satisfied	Moderately satisfied	Very satisfied
Overall satisfaction	☐	☐	☐	☐

21. Looking back, what do you most strongly associate with your time in the programme?

Open-ended Text; no response

22. Do you have any additional comments that are important for the evaluation?

Open-ended Text; no response
